# 3D Printing of Continuous Basalt Fiber-Reinforced Composites: Characterization of the In-Plane Mechanical Properties and Anisotropy Evaluation

**DOI:** 10.3390/polym16233377

**Published:** 2024-11-30

**Authors:** Marco Zanelli, Giulia Ronconi, Nicola Pritoni, Andrea D’Iorio, Monica Bertoldo, Valentina Mazzanti, Francesco Mollica

**Affiliations:** 1 Department of Engineering, University of Ferrara, Via Saragat 1, 44122 Ferrara, Italy; marco.zanelli@unife.it (M.Z.); giulia.ronconi@unife.it (G.R.); nicola.pritoni@unife.it (N.P.); francesco.mollica@unife.it (F.M.); 2 Department of Chemical, Pharmaceutical and Agricultural Sciences, University of Ferrara, Via L. Borsari 46, 44121 Ferrara, Italy; andrea.diorio@unife.it (A.D.); monica.bertoldo@unife.it (M.B.)

**Keywords:** natural fiber, fused deposition modeling, co-extruded continuous fiber, Polyamide 12, Anisoprint Composer

## Abstract

Usage of continuous fibers as a reinforcement would definitely increase the mechanical properties of 3D-printed materials. The result is a continuous fiber-reinforced composite obtained by additive manufacturing that is not limited to prototyping or non-structural applications. Among the available continuous reinforcing fibers, basalt has not been extensively studied in 3D printing. This material is attractive due to its natural origin, good mechanical properties, impact strength, and high chemical and thermal resistance. In this work, a continuous basalt fiber co-extruded composite obtained by fused filament fabrication was characterized both thermally and mechanically, concerning the in-plane tensile properties. The degree of anisotropy of the material was also assessed, both qualitatively and quantitatively. The 3D-printed composite showed longitudinal properties, which were 15 times higher than the pure matrix, thus meeting structural requirements. On the other hand, transverse and shear properties were much lower than longitudinal ones, thus leading to a strongly anisotropic material. This was also confirmed by the anisotropy evaluation that was performed numerically and graphically using an innovative approach. This behavior affects the design of 3D-printed parts; thus, an optimized continuous fiber deposition is necessary for structural applications.

## 1. Introduction

Fused filament fabrication (FFF) is a 3D-printing technique that allows to produce plastic parts by depositing material layer by layer. Despite every thermoplastic (TP) material can potentially be used, there are some polymers that are more common, such as acrylonitrile butadiene styrene (ABS), polylactic acid (PLA), and polyamides (PAs). FFF allows one to obtain parts that can have complex shapes, with mechanical properties that can be tailored by choosing suitable printing parameters [1,2].

For instance, the raster angle is an important parameter, which represents the line orientation during the material deposition in a specific 3D-printed layer, and clearly influences the mechanical properties [3]. Indeed, it is expected that due to molecular orientation induced by the line deposition, the 3D-printed part is stiffer and stronger when loaded parallel to the raster angle than in any other direction. This is especially true in the case of short-fiber-reinforced TPs, which are often employed for increasing the mechanical properties of printed materials with the goal of achieving structural characteristics [3].

In order to increase the mechanical properties of printed materials even further, in recent years, a few 3D printers that are capable of printing continuous fiber-reinforced TPs have appeared on the market. Even though these machines are still in their initial stage of development, their potential is definitely very attractive, as they allow for the relative freedom of a shape typical of 3D printing combined with the mechanical properties offered by continuous fiber reinforcement.

One possibility of depositing continuous fiber reinforcement is through the presence of two extruding nozzles, one for the standard TP, and the other one for the continuous fiber. The whole process, though, requires an ad hoc-developed slicing software that is sufficiently sophisticated that can manage the deposition from both nozzles successfully.

Carbon fiber provides the highest mechanical properties, and already a certain number of papers that appeared in the recent literature are devoted to the characterization of continuous carbon fiber-reinforced 3D-printed materials. In particular, [4,5] studied the mechanical properties of continuous carbon fiber-reinforced PLA, while [6,7,8] used poly-(ethylene terephthalate—glycol-modified) (PETG) as the matrix and [9,10,11,12] used PA. On the other hand, the environmental impact of such fibers is quite relevant due to their fossil origin; therefore, reinforcement fibers of natural origin are increasingly studied and also employed in various industrial applications, due to their attractive eco-sustainability [13,14,15].

Basalt fibers are an interesting type of natural fibers. Basalt can be classified as a sustainable material, because it does not require additives for its production and during the technological process, there are no emissions of toxic substances. Through melt drawing and solution spinning, this material can be used in the form of powder, short and continuous fibers, in a wide range of application areas [16,17]. Although they do not reach the same mechanical properties as carbon fibers, they possess strength and stiffness that are similar to glass fibers; therefore, they can be used as an environmentally friendly substitute [18]. Moreover, they are superior to glass in terms of heat and acoustic damping and outstanding vibration isolation, corrosion resistance, price and can operate in a sufficiently wide thermal range, so that they can even be used as a thermal shield [19].

To the best of the authors’ knowledge, the only papers dealing with continuous basalt fiber-reinforced 3D-printing composites are [20,21]. In both articles, the composites were tested in tension and bending but only in the longitudinal direction. 3D-printed composites have low transverse and shear properties [3,9,22,23], but the longitudinal ones are definitely higher; thus, a complete mechanical characterization as an anisotropic material is required.

The aim of this article is to characterize the in-plane properties both in stiffness and strength of a 3D-printed co-extruded composite made of a continuous basalt fiber-reinforced PA12. Its anisotropic behavior was then evaluated both qualitatively and quantitatively. The results are compared with those obtained from the characterization of two standard TP materials, an unfilled PA12 and a PA12 reinforced with 10 wt.% short carbon fiber.

## 2. Mechanical Behavior Modeling of 3D-Printed Parts

### 2.1. Mathematical Modeling

As is well known from the scientific literature [22,23], reinforced 3D-printed polymeric materials can be successfully modeled using theories that are typical of composites. In fact, a layer printed with a specific raster angle is anisotropic in the same way as a unidirectional composite lamina; hence, it can be modeled using lamina macromechanics [24,25].

**Figure 1:**
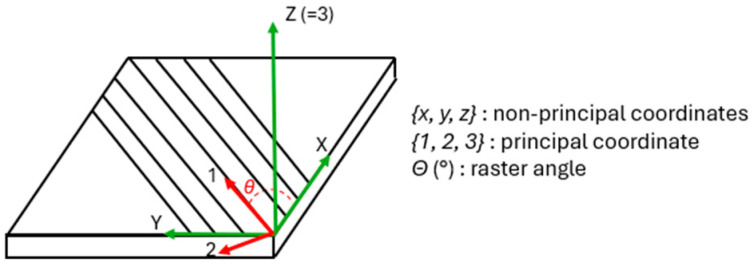
The lamina and its coordinate systems.

Two right-handed coordinate systems are normally considered: the first one, indicated as x, y, and z in Figure 1, is a convenience coordinate system that is often related to the external loading, while $x_{1}$, $x_{2}$, and $x_{3}$ are related to material symmetry. In particular, axes $x_{3}$ and z coincide, while $x_{1}$ is rotated with respect to x by the raster angle ϑ. Since a 3D-printed layer can be thought of as an orthotropic elastic material loaded in plane stress, four constants are necessary to represent its behavior, namely $E_{1}$, $E_{2}$, $G_{12}$, and $\nu_{12}$. $E_{1}$ and $E_{2}$ are Young’s moduli in the longitudinal and transverse directions, and $G_{12}$ is the in-plane shear modulus, while $\nu_{12}$ is Poisson’s ratio. Concerning strength, it is assumed that only the three independent material constants $S_{L}$, $S_{T}$, and $S_{LT}$ describe the mechanical behavior and these are longitudinal, transverse, and shear strengths, respectively.

Following the usual notation, the layer stiffness in the $x_{1}$, $x_{2}$, and $x_{3}$ coordinate system is the matrix $\left[Q\right]$ defined as follows:

(1) $$\left\{\sigma \right\}=\left[Q\right]\left\{\varepsilon \right\}\mathrm{or}\;\left\{\begin{array}{c} \begin{array}{c} \sigma_{1}\\ \sigma_{2} \end{array}\\ \sigma_{6} \end{array}\right\}=\left[\begin{array}{ccc} Q_{11} & Q_{12} & 0\\ Q_{12} & Q_{22} & 0\\ 0 & 0 & Q_{66} \end{array}\right]\left\{\begin{array}{c} \begin{array}{c} \varepsilon_{1}\\ \varepsilon_{2}\; \end{array}\\ \varepsilon_{6} \end{array}\right\}$$

where $\sigma_{1}$ and $\sigma_{2}$ are the normal stresses and $\sigma_{6}$ is the in-plane shear stress. In analogy, $\varepsilon_{1}$ and $\varepsilon_{2}$ are the normal strains and $\varepsilon_{6}$ is the in-plane engineering shear strain. The stiffness matrix components are

(2) $$Q_{11}=\frac{E_{1}}{1-\nu_{12}\nu_{21}},\;Q_{22}=\frac{E_{2}}{1-\nu_{12}\nu_{21}}\;,\;\;Q_{12}=\frac{{\nu_{12}E}_{2}}{1-\nu_{12}\nu_{21}}\;,\;Q_{66}=G_{12}\;\mathrm{with}\;\nu_{21}=\nu_{12}\frac{E_{2}}{E_{1}}$$

The stiffness matrix changes its representation in the x, y, and z coordinate system as follows:

(3) $$\left\{\begin{array}{c} \sigma_{x}\\ \sigma_{y}\\ \sigma_{s} \end{array}\right\}=\left[\begin{array}{ccc} Q_{11}^{\vartheta } & Q_{12}^{\vartheta } & Q_{16}^{\vartheta }\\ Q_{12}^{\vartheta } & Q_{22}^{\vartheta } & Q_{26}^{\vartheta }\\ Q_{16}^{\vartheta } & Q_{26}^{\vartheta } & Q_{66}^{\vartheta } \end{array}\right]\left\{\begin{array}{c} \varepsilon_{x}\\ \varepsilon_{y}\\ \varepsilon_{s} \end{array}\right\}$$

where $\varepsilon_{x}$ and $\varepsilon_{y}$ and $\sigma_{x}$ and $\sigma_{y}$ are the normal strains and stresses, while $\varepsilon_{s}$ and $\sigma_{s}$ are the engineering shear strain and stress in the x−y plane. The stiffness matrix [$\;Q^{\vartheta }$] depends on ϑ and on matrix $\left[Q\right]$ through the following:

(4) $$\begin{array}{l} Q_{11}^{\vartheta }=Q_{11}\cos^{4}\vartheta +Q_{22}\sin^{4}\vartheta +2\left(Q_{12}+{2Q}_{66}\right)\cos^{2}\vartheta \sin^{2}\vartheta \\ Q_{22}^{\vartheta }=Q_{11}\sin^{4}\vartheta +Q_{22}\cos^{4}\vartheta +2\left(Q_{12}+{2Q}_{66}\right)\cos^{2}\vartheta \sin^{2}\vartheta \\ Q_{12}^{\vartheta }=\left(Q_{11}+{Q_{22}-4Q}_{66}\right)\cos^{2}\vartheta \sin^{2}\vartheta {+Q}_{12}\left(\cos^{4}\vartheta +\sin^{4}\vartheta \right)\\ Q_{16}^{\vartheta }=\left(Q_{11}-{Q_{12}-2Q}_{66}\right)\cos^{3}\vartheta \sin\vartheta -\left(Q_{22}-{Q_{12}-2Q}_{66}\right)\sin^{3}\vartheta \cos\vartheta \\ Q_{26}^{\vartheta }=\left(Q_{11}-{Q_{12}-2Q}_{66}\right)\cos\vartheta \sin^{3}\vartheta -\left(Q_{22}-{Q_{12}-2Q}_{66}\right)\sin\vartheta \cos^{3}\vartheta \\ Q_{66}^{\vartheta }=\left(Q_{11}+{Q_{22}-2Q}_{12}\right)\cos^{2}\vartheta \sin^{2}\vartheta +Q_{66}{\left(\cos^{2}\vartheta -\sin^{2}\vartheta \right)}^{2} \end{array}$$

### 2.2. Quantification of Stiffness Anisotropic Behavior

An alternative form of Equation (4) was introduced in the literature [25,26]:

(5) $$\begin{array}{l} Q_{11}^{\vartheta }=U_{1}+U_{2}\cos\left(2\vartheta \right)+U_{3}\cos\left(4\vartheta \right)\\ Q_{22}^{\vartheta }=U_{1}-U_{2}\cos\left(2\vartheta \right)+U_{3}\cos\left(4\vartheta \right)\\ \begin{array}{l} Q_{12}^{\vartheta }=U_{4}-U_{3}\cos\left(4\vartheta \right)\\ Q_{16}^{\vartheta }=\frac{U_{2}}{2}\sin\left(2\vartheta \right)+U_{3}\sin\left(4\vartheta \right)\\ \begin{array}{l} Q_{26}^{\vartheta }=\frac{U_{2}}{2}\sin\left(2\vartheta \right)-U_{3}\sin\left(4\vartheta \right)\\ Q_{66}^{\vartheta }=\frac{1}{2}\left(U_{1}-U_{4}\right)-U_{3}\cos\left(4\vartheta \right) \end{array} \end{array} \end{array}\mathrm{with}\;\begin{array}{l} U_{1}={\frac{1}{8}(3Q}_{11}+3Q_{22}+2Q_{12}+4Q_{66})\\ U_{2}={\frac{1}{2}(Q}_{11}-Q_{22})\\ \begin{array}{l} U_{3}={\frac{1}{8}(Q}_{11}+Q_{22}-2Q_{12}-4Q_{66})\\ U_{4}={\frac{1}{8}(Q}_{11}+Q_{22}+6Q_{12}-4Q_{66}) \end{array} \end{array}$$

As reported in [25,26], $U_{1}$ and $U_{4}$ are invariant quantities under a change of reference frame; therefore, they represent the isotropic contributions to material stiffness. The other two quantities, $U_{2}$ and $U_{3}$, are not invariant and hence they are the orthotropic contributions and have a direct physical meaning in terms of deviation from the isotropic behavior. In particular, $U_{2}$ is related to the difference between longitudinal and transverse properties, i.e., between the two Young’s moduli $E_{1}$ and $E_{2}$, while $U_{3}$ indicates the dependence of $G_{12}$ on $E_{1}$, $E_{2}$, and $\nu_{12}$.

**Figure 2:**
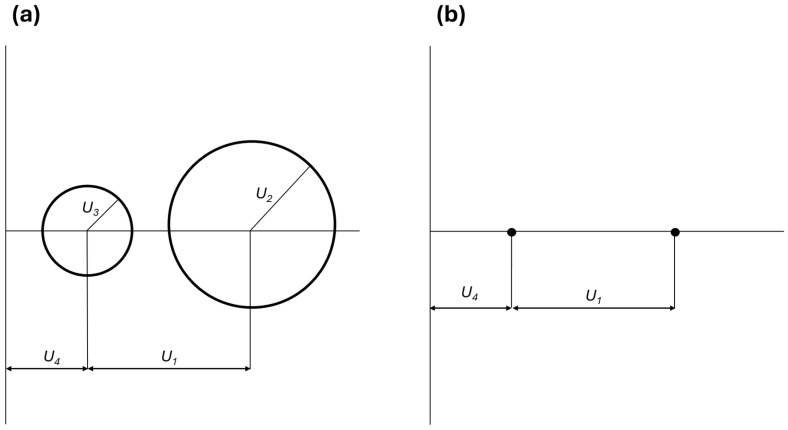
Generalized Mohr’s circles for (**a**) orthotropic lamina and for (**b**) isotropic lamina.

Tsai and Hahn [26] showed that it is convenient to represent the components of the stiffness matrix in the reference system x, y, and z (Equation (5)) through two generalized Mohr’s circles (Figure 2), based on $U_{1}$, $U_{2}$, $U_{3}$, and $U_{4}$. In their approach, the center of the first circle is placed on the abscissa axis at a distance $U_{4}$ from the origin and its radius is $U_{3}$. The center of the second circle is located at a distance $U_{1}$ from the center of the first circle and its radius is $U_{2}$.

This representation is convenient to evaluate graphically and qualitatively the degree of anisotropy of the in-plane stiffness. In fact, if the material is strongly anisotropic, the two circles will appear large with respect to their distance (Figure 2a). On the other hand, if the material is isotropic, $U_{1}\neq 0$ and $U_{2}=U_{3}=0$; thus, the circles will collapse to single points, and their distance is the only significant quantity (Figure 2b).

It could be useful to also assess anisotropy from a quantitative point of view. For a proper estimate, it is better if dimensionless parameters are used. Indeed, the orthotropic components $U_{2}$ and $U_{3}$ are intrinsically dependent on the stiffness properties of the material (Equation (5)); thus, they should be normalized with a reference stiffness measurement to obtain dimensionless quantities. In this work, $\frac{U_{2}}{U_{1}}$ and $\frac{U_{3}}{U_{1}}$ ratios were used, and $U_{1}$ is preferred to $U_{4}$ because it is a measure of material stiffness and can never be zero, since it is defined as a sum and not as a difference (like $U_{4}$). Moreover, these ratios represent the relative weight of the orthotropic components with respect to the isotropic one.

When these ratios are relatively large, the material has a pronounced anisotropy, while if the material is isotropic, they will be close to zero. Moreover, if $\frac{U_{2}}{U_{1}}$ is greater than $\frac{U_{3}}{U_{1}}$, anisotropy is mainly due to the difference between the longitudinal and transverse properties. On the opposite side, the anisotropic behavior is due to the shear properties.

## 3. Materials and Methods

### 3.1. Materials

The materials used in this study are listed in Table 1 together with the information declared in the technical data sheet. CFCPA is a thermoplastic filament based on unreinforced PA12, while SmoothPA is a thermoplastic filament, also based on PA12, but contains 10 wt.% short carbon fibers, and in the remainder of this paper will be indicated with SMPA. Finally, CBF is a thermoset-coated continuous basalt fiber. All materials were acquired from Anisoprint Sarl (Mondercange, Luxembourg).

### 3.2. Thermal Characterization Instruments and Methods

Differential scanning calorimetric analyses were performed on a DSC 8000, Perki-nElmer Inc. (Waltham, MA, USA) instrument equipped with an IntraCooler II cooling device and Pyris software (Version 13.3, PerkinElmer Inc., Waltham, MA, USA) for instrument control, data acquisition, and analyses. The instrument was calibrated for temperature and energy with high-purity indium and lead as standards. All tests were made in a nitrogen atmosphere with two thermal cycles at a heating/cooling rate of 10 °C/min between 30 and 250 °C. DSC was used to determine the crystalline fraction and the characteristic temperatures, such as the glass transition temperature $T_{g}$ and the melting temperature $T_{m}$. The crystalline fraction $\chi_{c}$, evaluated as a percentage, was obtained as follows:

(6) $$\chi_{c}=\frac{\Delta H_{m}}{\Delta H_{m0}(1-w_{t})}*100$$

where $\Delta H_{m}$ is the experimentally measured melting enthalpy, and $\Delta H_{m0}$ is the melting enthalpy of the fully crystalline polymeric matrix and for PA12, it was assumed to be equal to 245 J/g [27].

The fiber or filler mass fraction $w_{t}$ was measured through a thermogravimetric analysis or TGA (PerkinElmer TGA 4000 thermobalance. Waltham, MA, USA), which is useful to also evaluate the thermal stability of the filaments. The measurements were carried out on 5–10 mg of material under a nitrogen atmosphere (30 mL/min), from 30 to 900 °C at a heating rate of 10 °C/min.

Infrared spectroscopy analyses were accomplished with a PerkinElmer (Perkin-Elmer Inc., Norwalk, CT, USA) Spectrum 100 FT-IR spectrometer equipped with a Universal ATR accessory.

A CHNS/O analysis was accomplished by using a FLASH2000 instrument (Ther-moFisher Scientific, Waltham, MA, USA).

### 3.3. 3D-Printer

**Figure 3:**
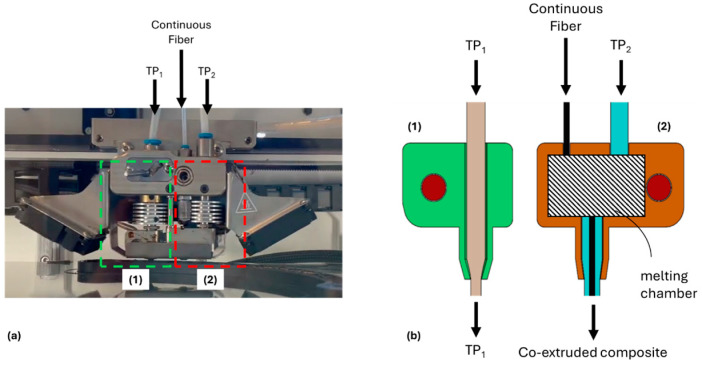
Printhead of Anisoprint Composer A3: (**a**) picture and (**b**) schematic representation.

All specimens were printed using a Composer A3 by Anisoprint. The printhead is equipped with two different extruders (Figure 3): The left one is a standard Bowden device with a 0.4 mm nozzle and receives the ordinary thermoplastic filament TP_1_. The right one is input with the continuous reinforcement fiber and another thermoplastic filament, TP_2_, that is molten within a melting chamber and co-extruded with the fiber through a 0.6 mm nozzle.

**Figure 4:**
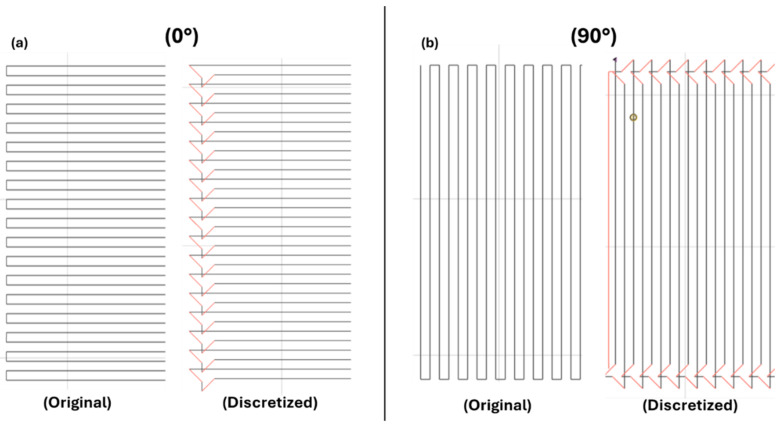
Original and discretized trajectories for (**a**) 0° and (**b**) 90° pattern.

The slicing software used is the open version of Aura 2.4.8, which allows for the management of the various printing parameters in both extruders, such as the fiber–matrix ratio and fiber orientation during deposition after the co-extrusion process. Curved trajectories and changes in direction are critical due to the stiffness of the continuous fiber, and this can induce weak points, poor aesthetics, the presence of voids, and dimensional inaccuracy. To limit these problems, Aura discretizes the original path to ensure that the fiber follows it as closely as possible (Figure 4) and moreover the printing speed is slowed down considerably at the critical points.

### 3.4. Specimen Preparation

CBF-reinforced PA12 specimens were obtained by co-extrusion using the right extruder of the Composer A3 printer. The acronym CBFcc, which stands for a CBF co-extruded composite, is used in this paper to refer to this material. Rectangular specimens were manufactured with 100% infill density in flat configuration and tested according to the ASTM D3039 and ASTM D3518 standards [28,29]. In particular, the former standard is used to measure longitudinal and transverse properties, by printing specimens at 0° and 90° raster angles, while the latter is used for shear characterization, and specimens were printed at ±45° raster angles. For completeness, CFCPA and SMPA are characterized in the same way, but were printed using the left extruder of the 3D printer.

All specimens were printed using a single line skirt to purge and prepare the extruder before printing, without external shells and top or bottom layers. The bed was heated at 65 °C and all polyamide-based filaments were dried at 80 °C for 24 h and the spools were stored in a passive dry box (Polymaker, Shanghai, China) containing silica salts during printing.

The specimen dimensions in terms of length, width, and thickness are listed in Table 2 for each material, together with the main printing parameters. From the thickness and the layer height, the number of layers can be estimated for each sample.

**Figure 5:**
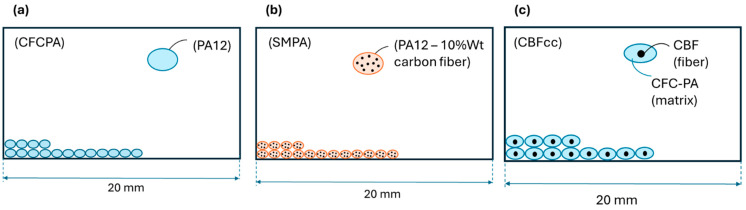
Representative section of specimens for (**a**) CFCPA, (**b**) SMPA, and (**c**) CBFcc.

**Figure 6:**
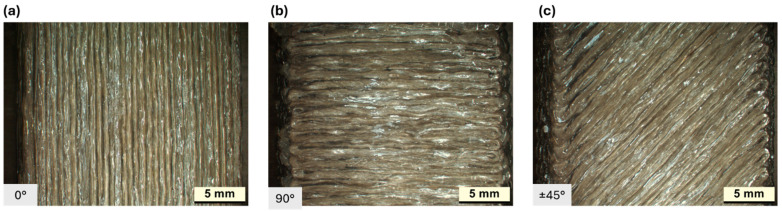
CBFcc fiber orientation at (**a**) 0°, (**b**) 90°, and (**c**) ±45°.

Notice that while CFCPA is an unreinforced thermoplastic, both SMPA and CBFcc are composites: they differ by the material of the reinforcing fibers (carbon for SMPA, basalt for CBFcc) and by the fiber length (short fiber for SMPA and continuous fibers for CBFcc). This peculiarity is well described in Figure 5. Figure 6 shows the fiber orientation for CBFcc, for the three raster angle orientations. It is possible to observe fiber bending at the edges, especially for 90° and ±45°.

### 3.5. Density and Void Measurements

Density was measured for the three filaments $\rho_{CBF}^{f},\rho_{CFCPA}^{f},{and\;\rho }_{SMPA}^{f}$ and for the tensile rectangular specimens before testing $\rho_{CBFcc}^{s},\rho_{CFCPA}^{s},{and\;\rho }_{SMPA}^{s}$, following ASTM D3171-22 [30]. Mass measurements were performed in all cases with a precision scale (AdventurerPro AV4102C, Pine Brook, NJ, USA, with ±0.01 g resolution). The cross-sectional area of the filaments was measured with a stereomicroscope (Leica MZ6, with Leica EC3 camera, Leica Camera AG, Wetzlar, Germany), on five different pieces of filament for each material. A caliper (±0.01 mm resolution) was used to measure the precise length of the filaments (about 50 mm) and the dimensions (length, width, thickness) of each specimen were obtained by averaging three different measurements at different points.

The density of the co-extruded composite $\rho_{CBFcc}^{f}$ is estimated using the rule of mixtures (ROM):

(7) $$\rho_{CBFcc}^{f}=\rho_{CBF}^{f}\phi_{CBF}+\rho_{CFCPA}^{f}\phi_{CFCPA},$$

where $\phi_{CBF}$ and $\phi_{CFCPA}$ are the volume fractions of the two materials, obtained by Aura during the print preview, respectively.

The void percentage of the specimens %v was evaluated as follows:

(8) $$\%v=\left(1-\frac{\rho_{x}^{s}}{\rho_{x}^{f}}\right)\cdot 100$$

where x is the considered material. The calculation of $\rho_{x}^{s}$ was performed for each orientation (0°, 90°, ±45°).

### 3.6. Tensile Property Measurements and Material Characterization

Tensile tests were carried out at room temperature at a crosshead speed of 2 mm/min using a universal testing machine (INSTRON 4467, INSTRON, Norwood, MA, USA) with a 30 kN load cell. The tests were performed on three samples per configuration (0°, 90°, ±45°), for a total of nine samples per material.

For all specimens, Digital Imaging Correlation, or DIC (DANTEC DYNAMICS, Skovlunde, Denmark), was used to measure both longitudinal and transverse strains. These were evaluated as an average over the analyzed area. The surface of the sample that was attached to the printing bed was painted with water-based ink, to ensure readability by the DIC cameras.

Young’s modulus $E_{1}$, Poisson’s ratio $\nu_{12}$, and longitudinal strength $S_{L}$ were obtained from 0° orientation, and Young’s modulus $E_{2}$ and transverse strength $S_{T}$ were evaluated from 90° specimens, while the shear modulus $G_{12}$ and shear strength $S_{LT}$ were evaluated from the ±45° ones.

Following ASTM D3039, $E_{1}$, $E_{2}$, and $\nu_{12}$ were estimated between 1000 *με* and 3000 *με*, while $G_{12}$ was calculated between 1500 *με* and 4000 *με*, as described in ASTM D3518. Strength values were evaluated as offset strengths, with the value of the offset strain being 2000 *με*, as described in both standards.

## 4. Results and Discussion

### 4.1. Processing Window

CFCPA melts at 168.6 ± 1.0 °C (peak maximum in the DSC thermogram) and its glass transition temperature is 64.9 ± 1.0 °C. The melting enthalpy is 27.5 J/g, corresponding to a crystallinity degree of 11.5%. By cooling at a constant rate of 10 °C/min, the polymer almost does not crystallize, and in the second heating cycle, a small melting peak corresponding to roughly 1% of crystallinity is detected. However, after melting the filament in a thermopress and letting the obtained dish freely cool down at room temperature, the crystalline phase developed with a degree comparable to the one in the pristine filament. SmoothPA melts at 160.3 ± 1.0 °C (the peak maximum in the DSC thermogram) and its glass transition temperature is 61.9 ± 1.0 °C. The melting enthalpy is 16.5 J/g, corresponding to a modest crystallinity degree of 7.5%. By cooling at a constant rate of 10 °C/min, the polymer does not crystallize; however, it does after melting in a thermopress and letting it cool in open air.

The DSC analysis of the CBF filament shows a detectable glass transition temperature at 76.5 °C, indicating the presence of a polymeric component, in agreement with the declaration in the technical data sheet. No melting peak was determined both in the first and second heating, denoting that the resin used for the coating of the basalt fiber is amorphous.

By the CHNS analysis of the CBF filament, only carbon and hydrogen were detected. In the infrared spectrum of the fibers , vibrational bands in the 3100–2800 cm^−1^ region due to $\upsilon_{C-H}$ of aromatic and aliphatic structures, a band at 1732 cm^−1^ due to $\upsilon_{C=O}$, two bands at 1580 and 1510 cm^−1^ due to $\upsilon_{C=C}$ of aromatic bonds, and a band at 1300 cm^−1^ assigned to $\upsilon_{C-O}$ of ester groups were observed. All these findings indicate that the thermosetting coating on the fiber is made by an aromatic polyester.

**Figure 7:**
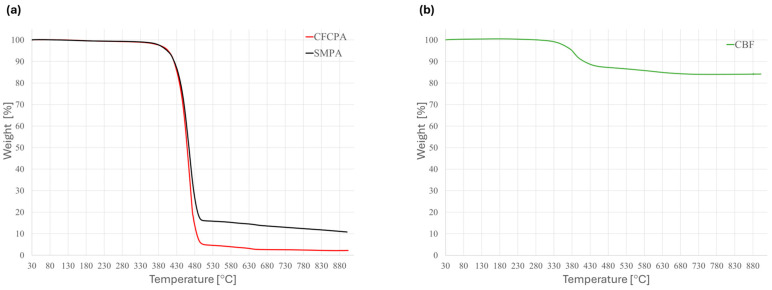
TGA plots for the three starting filaments: (**a**) CFCPA and SMPA, (**b**) CBF.

The thermal stability of the filaments was evaluated by the TGA analysis (Figure 7). The results obtained for SMPA and CFCPA show a similar onset of degradation at 435 °C, in good agreement with other data for PA12 filaments reported in the literature [31,32]. For both materials, the results agree with the material datasheet as reported by the producer (Tab 1). In particular, CFCPA shows a 2.2% residuum at 900 °C, which may indicate a small presence of mineral fillers. SMPA shows the same behavior, but the final residual mass is 10.8%, mostly due to the presence of the short carbon fiber reinforcement.

The CBF thermogram shows an onset of degradation at 358 °C with the main weight loss (12%) occurring in the 350–400 °C temperature range, as also observed in [33]. This step is attributed to the degradation of the organic polyester coating present on the basalt fibers used in this work (CBF). The presence of this fraction is confirmed by both CHNS and FT-IR analyses. While 358 °C is the temperature at which the sample lost 5% of its weight by heating at 10 °C/min, up to 280 °C, no other weight loss is observed up to 900 °C. Overall, the CBF filament shows 84.2% residual mass at 900 °C, which is attributed to the mass fraction of BF in CBF.

Notice that the temperature of 280 °C should not cause degradation even for longer heating times of both the inorganic and organic components of the CBF and thus it is assumed as the upper limit that must not be exceeded during co-extrusion to prevent fiber degradation. Indeed, for the preparation of a polymer-based composite, the presence of the coating was reported to be advantageous to promote adhesion at the fiber–matrix interface [34]. On the other hand, the minimum processing temperature must allow for filament melting and flowing. Based on the melting of SMPA and CFCPA (T_m_, 160 °C and 168 °C, respectively), processing must be performed at least above 180 °C. Indeed, typically PA12 is extruded by setting the processing temperature in the 185–270 °C temperature range [31,32].

### 4.2. Density and Void Results

The densities of the three starting filaments were measured, both for the two TP materials ($\rho_{CFCPA}^{f},\rho_{SMPA}^{f}$) and for the continuous basalt reinforcement fiber ($\rho_{CBF}^{f}$). The density of the co-extruded composite material ($\rho_{CBFcc}^{f}$) was calculated following Equation (7), using the volumetric fractions $\phi_{CFCPA}$= 0.66 and $\phi_{CBF}$= 0.34. The values are shown in Table 3.

CBF density is higher than the other materials due to the presence of basalt fiber, and SMPA density is slightly higher than CFCPA because of the carbon fibers, while CBFcc density is intermediate due to the co-extrusion process.

These data allow for the calculation of the void percentage according to Equation (8), and the corresponding values are listed in Table 4. It is important to note that the void percentage is heavily dependent on the raster angle of the specimens; therefore, the results are presented also in terms of this parameter. In addition to this external placement of voids, there is an inherent void generation in FFF 3D printing, whereby the deposited lines have an elliptical shape (Figure 5) and voids are positioned between a deposited line and the adjacent one.

For each material, the longitudinal configuration has a lower void percentage (Figure 6), and this is mainly due to a reduced number of direction changes during printing, as described in Figure 4. To achieve the pattern at 90° and ±45°, more changes in direction are required, resulting in a slight increase in voids, and this is true especially for CFCPA and SMPA. For CBFcc, the highest percentage of voids is found in samples at ±45°, while at 90°, it is very low. This is due to the fact that to manufacture these specimens, it was necessary to reduce the z-offset during printing, which resulted in a greater compaction of the material and a limited presence of voids.

Void percentages are around 5% and, in some cases, even lower. For all samples, there is a low standard deviation, and this indicates good printing quality and process repeatability.

The CBF filament is made from continuous basalt fiber and TS coating. The basalt volume fraction of the filament $\phi_{BAS}$ can be evaluated from the following equations:

(9) $$\frac{1}{\rho_{CBF}^{f}}=\frac{\Psi_{BAS}}{\rho_{BAS}}+\frac{1-\Psi_{BAS}}{\rho_{TS}}$$

(10) $$\rho_{CBF}^{f}=\rho_{BAS}\phi_{BAS}+\rho_{TS}(1-\phi_{BAS})$$

where $\Psi_{BAS}$ is the basalt mass fraction equal to 0.842, evaluated from the TGA analysis; $\rho_{BAS}$ is the density of raw basalt, which from the literature equals 3 g/cm^3^ [35]; and $\rho_{CBF}^{f}$ is the density of the basalt filament, which was calculated previously. The only unknowns are the density of the TS coating, $\rho_{TS}$, and $\phi_{BAS}$, which result in 0.59 g/cm^3^ and 0.51, respectively. Taking this value into account, the effective volume fraction of basalt fiber of CBFcc samples is finally 0.18, which corresponds to a mass fraction of 0.41.

### 4.3. Tensile Test Results

#### 4.3.1. Mechanical Characterization

**Figure 8:**
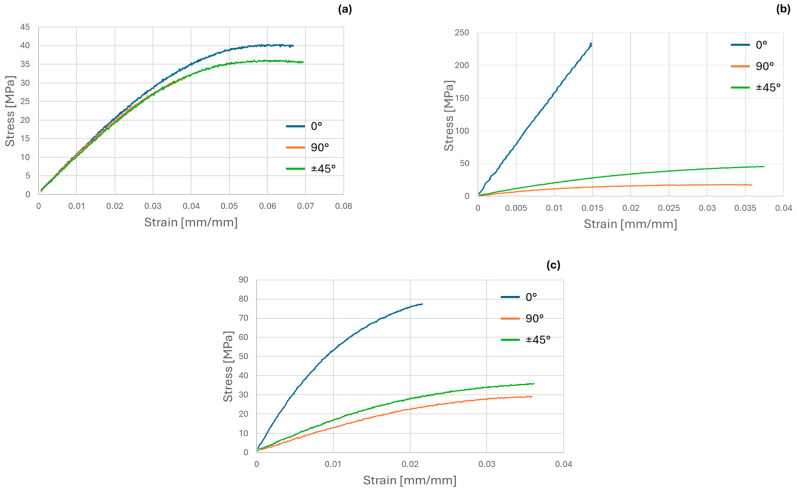
Stress–strain curves at different orientations for (**a**) CFCPA, (**b**) CBFcc, and (**c**) SMPA.

Representative stress–strain curves of the different configurations are provided in Figure 8a for CFCPA, in Figure 8b for CBFcc, and in Figure 8c for SMPA.

Figure 8a shows an elasto-plastic behavior, and the three curves overlap in the initial linear elastic region, regardless of the printing orientation; therefore, CFCPA stiffness behavior can be assumed as isotropic. On the other hand, there is a slight difference in terms of yield stress, but this is not enough to conclude that the material is anisotropic in terms of strength; therefore, it can be reasonably considered completely isotropic.

For CBFcc, the development of the curves is completely different (Figure 8b). The 0° curve has significantly higher stiffness and strength than 90° and ±45°; therefore, this material has a markedly anisotropic behavior. Moreover, the material is brittle in the longitudinal direction while it is ductile in the other two.

In the case of SMPA (Figure 8c), the three curves still show elasto-plastic behavior, but quantitatively the strength and stiffness values are greater at 0° than in the other two orientations. A certain degree of anisotropy in terms of stiffness and strength is clearly visible.

Table 5 shows the four elastic constants required for stiffness characterization for the three materials. Comparing the matrix (CFCPA) and the co-extruded composite (CBFcc), the latter has higher mechanical properties, especially $E_{1}$, which increases by more than an order of magnitude, while concerning $E_{2}$ and $G_{12}$, there is only a slight increase. Indeed, in the 0° configuration, the fibers are placed in the same direction as the applied load. Thus, fibers are the load-bearing component of the composite. At 90° and ±45° orientations, fibers have a less favorable orientation; thus, the behavior depends by a greater amount on the matrix. SMPA has a longitudinal modulus that is about five times that of the transverse modulus, in agreement with Figure 8c above. Poisson’s ratio is about 0.45, for all materials.

From the tensile tests, the strength values were also evaluated and are shown in Table 6. The strength behavior has the same trend as the stiffness. The values for CFCPA are about the same in the longitudinal and transverse directions, while the shear strength value is about half, in agreement with the hypothesis of isotropy.

For CBFcc, $S_{L}$ is approximately 20 times larger than $S_{LT}$ and $S_{T}$. The transverse strength for CBFcc is lower than that of the matrix (CFCPA) and this is in agreement with the literature [36], since it is expected that for unidirectional composites, fibers weaken the material in the transverse direction. This does not appear in the case of shear strength but notice that there is a considerably higher result dispersion for CBFcc (4.91 MPa over 15.37 MPa). This is a clear indication of a high degree of anisotropy of CBFcc with respect to the isotropic matrix (CFCPA), and also in the case of strength.

The strength values for SMPA at 0° are higher than those at 90° and ±45°, but still of the same order of magnitude, and again there is a slight degree of anisotropy.

For all stiffness and strength characteristics, a very low standard deviation of less than 5% from the average reference value can be observed, which indicates good printing quality and process repeatability.

#### 4.3.2. Stiffness Anisotropy

**Figure 9:**
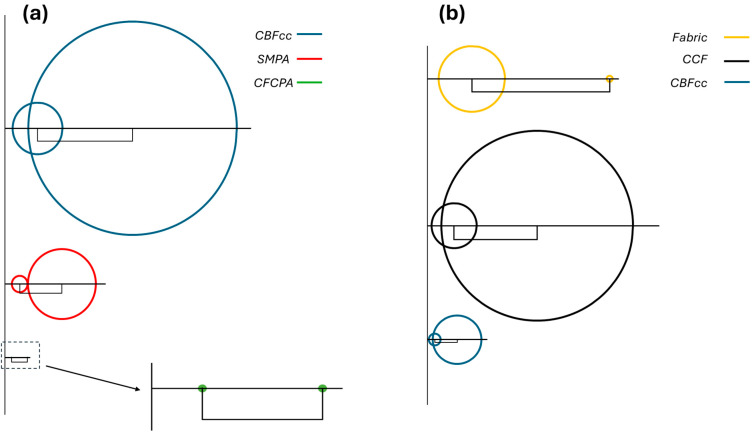
Comparison of generalized Mohr circles referring to CBFcc: (**a**) with respect to CFCPA and SMPA, (**b**) with respect to CCF [37] and fabric [38].

As described in Section 2.2, we propose to quantify the anisotropy of the in-plane stiffness properties for the tested materials. Once $E_{1}$, $E_{2}$, $G_{12}$, and $\nu_{12}$ are known, the anisotropic behavior can be evaluated graphically and qualitatively from the generalized Mohr’s circles (Figure 9), and quantitatively from the ratios $\frac{U_{2}}{U_{1}}$ and $\frac{U_{3}}{U_{1}}$ (Table 7).

Referring to the generalized Mohr’s circles for CFCPA (Figure 9a), the radii ($U_{2}$, $U_{3}$) are very small compared to the distance between the centers ($U_{1}$), meaning that the orthotropic components are much smaller than the isotropic one. This behavior is also confirmed quantitatively by both ratios, which are very close to zero, indicating that the material is substantially isotropic.

Looking at the other two materials (Figure 9a), both the distance between the centers and the radii increase with respect to CFCPA, denoting that the mechanical properties are higher. Moreover, the proportion of the orthotropic components with respect to the isotropic one increases from CFCPA to SMPA and even more to CBFcc (Table 7). This can be easily seen by the size of the radii compared to the distance of the centers: for SMPA, the circles are very close, while they even intersect for CBFcc.

This trend indicates that the behavior is increasingly anisotropic, with a stronger degree in the case of CBFcc than SMPA, since both ratios increase by 35%.

In addition, both anisotropic ratios increase in comparison with CFCPA, to a larger extent in the case of CBFcc. For both the latter and SMPA, the major contribution is made by $\frac{U_{2}}{U_{1}}$, so anisotropy is mainly due to the difference between the longitudinal and transverse properties.

For completeness and in order to have a better understanding of the anisotropy estimate through generalized Mohr circles, two other materials taken from the literature were compared with CBFcc (Figure 9b, Table 7). The first one is a 3D-printed composite reinforced with unidirectional continuous carbon fiber (CCF) [37] and the second one is an epoxy-based composite reinforced with a [0°/90°] carbon fabric [38].

The circles of CCF are larger and more widely spaced than those of CBFcc, because carbon fibers are much stiffer than basalt. On the other hand, looking at the anisotropy ratios of CCF and CBFcc, the corresponding ones are basically identical and this shows that CCF is anisotropic in the same way as CBFcc. The trend is the same as before when comparing SMPA with CBFcc, and again the left circle is smaller than the right circle.

The generalized Mohr circles of the fabric are completely different from the CBFcc, as the two circles are widely spaced, with respect to the size of the radii, and the left one is larger than the right one. The type of anisotropy is completely different because reinforcing fibers are present in the longitudinal and transverse directions in equal amounts. Since the ratio $\frac{U_{3}}{U_{1}}$ is approximately 10 times of $\frac{U_{2}}{U_{1}}$, anisotropy is mainly due to the shear properties.

## 5. Conclusions and Future Developments

In this paper, three different materials were 3D-printed with FFF technology and tested in simple tension to obtain a complete characterization of the in-plane properties and an evaluation of their anisotropy.

A low percentage of voids (6.7% maximum) was achieved for all materials, which was allowed by the good setting of the 3D printer, and with a final effective volume fraction of basalt fiber of only 18% for CBFcc.

For the co-extruded composite (CBFcc), the use of continuous basalt fiber increases longitudinal stiffness (~18 GPa) and strength (~200 MPa) by more than an order of magnitude compared to the matrix (CFCPA). On the other hand, the transverse and shear properties are very low for CBFcc and comparable to CFCPA. CBFcc has a marked anisotropy, while the matrix shows isotropic behavior. SMPA is a commercial filament reinforced with 10 wt.% short carbon fiber, chosen for convenience because it has the same matrix (PA12) as the co-extruded composite and is a popular material in FFF 3D printing. Comparing the mechanical properties, CBFcc has longitudinal properties about 3.5 times larger than SMPA, while the shear and transverse properties follow the same trend as the previous comparison made between the co-extruded composite and the neat matrix. The different types of reinforcing fibers must be taken into account: carbon fibers in SMPA have higher mechanical properties than basalt, but they are short; thus, the overall performance of SMPA is lower than CBFcc, which is reinforced by continuous basalt fibers.

The anisotropic behavior of these 3D-printed composites was also evaluated using the method proposed by Tsai [26]. This has proven to be an efficient means of assessing stiffness anisotropy directly and quickly, both graphically via generalized Mohr circles and numerically via two non-dimensional quantities. The increasing impact of the orthotropic component on the isotropic one can be observed moving from an unfilled material to a short-fiber-reinforced one and then to a material with a continuous fiber as reinforcement. This novel method of interpreting anisotropy is generalizable and applicable to any material, as long as it is orthotropic and loaded in plane stress state.

For CBFcc, the longitudinal properties meet structural requirements, but the transverse and shear properties are not completely satisfactory. The optimization of the fiber deposition direction, as a function of the expected external loads, can be a possible strategy to make the most out of the directionality of the material properties. Another possibility to increase transverse and shear properties can be to perform post-treatments [39], and this may also be useful in decreasing the void percentage.

## Figures and Tables

**Table 1:** Material description from technical data sheets.

Name	Material	Diameter [mm]	Fiber Mass Fraction [%]	Young’s Modulus [GPa]	Tensile Strength [MPa]
CFCPA	PA12	1.75	-	1.25	41.10
SMPA	short carbon fiber-filled PA12	1.75	10	5.82	71.80
CBF	TS-coated basalt fiber	0.28	65 ÷ 75	50	1786

**Table 2:** Characteristics and printing parameters of samples for the three materials.

Characteristic	CFCPA	SMPA	CBFcc
Sample dimensions (mm)	200 × 20 × 4	200 × 20 × 4	200 × 20 × 2.4
Layer height (mm)	0.2	0.2	0.3
Line width (mm)	0.4	0.4	0.6
Extrusion temperature (°C)	250	265	250
Printing speed (mm/s)	40	40	30

**Table 3:** Densities for the three starting filaments; values in parentheses represent standard deviation, and that calculated for the co-extruded composite.

Filament Density (g/cm^3^)			Co-Extruded Density (g/cm^3^)
$\rho_{CFCPA}^{f}$	$\rho_{SMPA}^{f}$	$\rho_{CBF}^{f}$	$\rho_{CBFcc}^{f}$
0.99 (0.002)	1.01 (0.001)	1.82 (0.07)	1.28

**Table 4:** Void percentage for all configurations; values in parentheses represent standard deviation.

	*% Voids*		
Material	0°	90°	±45°
CFCPA	3.5 (0.7)	5.2 (0.2)	5.6 (0.4)
SMPA	2.9 (1.3)	6.7 (1.2)	6.2 (0.3)
CBFcc	1.4 (0.2)	2.8 (0.4)	5.7 (0.8)

**Table 5:** Elastic properties for all materials; values in parentheses represent standard deviation.

Material	$E_{1}$ (GPa)	$E_{2}$ (GPa)	$\nu_{12}$	$G_{12}$ (GPa)
CFCPA	1.11 (0.05)	1.07 (0.03)	0.41 (0.02)	0.35 (0.05)
CBFcc	17.96 (0.67)	1.43 (0.03)	0.48 (0.06)	0.49 (0.02)
SMPA	6.6 (0.1)	1.26 (0.04)	0.43 (0.01)	0.47 (0.06)

**Table 6:** Strength properties for all materials; values in parentheses represent standard deviation.

Material	$S_{L}$ (MPa)	$S_{T}$ (MPa)	$S_{LT}$ (MPa)
CFCPA	18.15 (1.14)	18.61 (1.88)	9.01 (0.46)
CBFcc	222.27 (11.87)	12.32 (0.63)	15.37 (4.91)
SMPA	54.33 (1.9)	20.73 (1.27)	12.46 (0.6)

**Table 7:** Orthotropic fractions for the five materials.

	Printed Materials			Literature References	
Ratio	CFCPA	SMPA	CBFcc	CCF [37]	Fabric [38]
$\frac{U_{2}}{U_{1}}$	0.02	0.81	1.10	1.15	0.02
$\frac{U_{3}}{U_{1}}$	0.02	0.19	0.26	0.27	0.24

## Data Availability

The data are contained within the article.
